# Constraints to Genetic Exchange Support Gene Coadaptation in a Tripartite RNA Virus

**DOI:** 10.1371/journal.ppat.0030008

**Published:** 2007-01-26

**Authors:** Fernando Escriu, Aurora Fraile, Fernando García-Arenal

**Affiliations:** 1 Departamento de Biotecnología, Universidad Politécnica de Madrid, Madrid, Spain; 2 Centro de Biotecnología y Genómica de Plantas, Universidad Politécnica de Madrid, Madrid, Spain; Pennsylvania State University, United States of America

## Abstract

Genetic exchange by recombination, or reassortment of genomic segments, has been shown to be an important process in RNA virus evolution, resulting often in important phenotypic changes affecting host range and virulence. However, data from numerous systems indicate that reassortant or recombinant genotypes could be selected against in virus populations and suggest that there is coadaptation among viral genes. Little is known about the factors affecting the frequency of reassortants and recombinants along the virus life cycle. We have explored this issue by estimating the frequency of reassortant and recombinant genotypes in experimental populations of *Cucumber mosaic virus* derived from mixed infections with four different pairs of isolates that differed in about 12% of their nucleotide sequence. Genetic composition of progeny populations were analyzed at various steps of the virus life cycle during host colonization: infection of leaf cells, cell-to-cell movement within the inoculated leaf, encapsidation of progeny genomes, and systemic movement to upper noninoculated leaves. Results indicated that reassortant frequencies do not correspond to random expectations and that selection operates against reassortant genotypes. The intensity of selection, estimated through the use of log-linear models, increased as host colonization progressed. No recombinant was detected in any progeny. Hence, results showed the existence of constraints to genetic exchange linked to various steps of the virus life cycle, so that genotypes with heterologous gene combinations were less fit and disappeared from the population. These results contribute to explain the low frequency of recombinants and reassortants in natural populations of many viruses, in spite of high rates of genetic exchange. More generally, the present work supports the hypothesis of coadaptation of gene complexes within the viral genomes.

## Author Summary

The exchange of genomic regions between viral strains or species is an important process in virus evolution, resulting often in dramatic changes in virulence and host range and in the emergence of new viral diseases. In spite of its potential importance, little is known about what factors affect the frequency of the resulting new genotypes in virus populations. We explore this issue using *Cucumber mosaic virus,* a plant virus with a tripartite RNA genome. Experimental populations were derived from mixed infections with different strains and were analyzed at different moments during host colonization. Results showed the existence of constraints to genetic exchange linked to various steps of the virus life cycle. These results contribute to explain the often low frequency of recombinant and reassortant genotypes in natural populations of many viruses, in spite of high rates of genetic exchange, and support the hypothesis of coadaptation of gene complexes within the viral genomes.

## Introduction

Genetic exchange is, with mutation, a primary source of genetic variation and plays an important role in virus evolution. Viruses possess mechanisms for genetic exchange that make their reproduction “just as sexual as that in eukaryotes” [1]: whenever different genetic variants replicate in the same cell, genetic exchange can occur by recombination of genome regions that are switched between nucleotide strands, or by reassortment of complete genome segments in viruses with segmented genomes. Genetic exchange results in novel genetic combinations that could have important phenotypic effects. It has been documented repeatedly that genetic exchange can result in dramatic changes in the properties of the viruses, and recombinant and reassortant genotypes have been associated often with host range expansion, with host switches, or with increased pathogenicity. An outstanding example is the reassortment between avian and human strains of influenza, resulting in novel viruses with pandemic potential, which is responsible for the most serious respiratory disease pandemic in humans; but other examples abound for both animal and plant viruses (e.g., [2–11]). In addition, genetic exchange may counterbalance the effect of deleterious mutation accumulation in virus populations [12], as initially shown by the classical work on bacteriophage Ø6, which showed that reassortment opposed the progress of mutational load in the virus populations [13,14]. In spite of its potential importance, rates of genetic exchange in viruses have been seldom analyzed (e.g., [15]), and little is known about how factors related to the virus life cycle or to the environment may affect the frequency of the resulting new genotypes in the virus population [16,17]. We have addressed the analysis of the factors that determine the frequency of reassortant and recombinant genotypes in virus populations, using *Cucumber mosaic virus* (CMV) as an experimental system.

CMV (genus *Cucumovirus,* family *Bromoviridae*) is a plant virus with a messenger-sense, single-stranded, three-segmented RNA genome. Each genome segment is encapsidated separately in an isometric particle. RNA1 and RNA2 encode proteins 1a and 2a, respectively, which are part of the virus replicase. RNA2 also encodes protein 2b, in a second open reading frame (ORF) overlapping that for protein 2a, which is a suppressor of the post-transcriptional gene-silencing defense of the host plant. RNA3 has two ORFs separated by a noncoding intergenic region, the 5′-most of which encodes the 3a movement protein, needed for cell-to-cell movement of the virus in the infected host. The second ORF of RNA3 encodes the coat protein (CP), which besides its structural function, is required for cell-to-cell and systemic movement and for vector transmission. CMV has a very broad host range, is transmitted in a nonpersistent manner by many species of aphids, and is found worldwide as the causal agent of economically important epidemics in many vegetable, fruit, and fodder crops (see [18] for a review). CMV isolates have been classified into three subgroups (named IA, IB, and II) according to sequence similarity between their genomic RNA3 [19]. Viable reassortants and recombinants can be obtained between CMV isolates belonging to subgroups IA, IB, and II [18], and on the basis of phylogenetic analyses it has been proposed that reassortment of genomic segments has played an important role in the evolution of CMV and has contributed to the high genetic diversity found among CMV strains [20]. Both reassortants and recombinants between CMV isolates belonging to different subgroups have been reported to occur in nature, but analyses of the genetic structure of CMV field populations have shown that reassortants and most recombinants were present at low frequency, and data indicated that they were at a selective disadvantage [21,22].

We have analyzed the frequency of reassortant and recombinant genotypes in plants double infected with CMV isolates from subgroups IA and IB. Analyses were done at various stages during colonization of the host plant by the virus, in order to dissect the role of different steps in the virus life cycle in the fate of the new genotypes. Results show that as plant colonization progresses, selection for particular gene combinations increases and the frequency distribution of the various possible genotypes departs more and more from random. Selection operates against genotypes with heterologous gene combinations resulting from genetic exchange between the parental strains, supporting the hypothesis of coadapted gene complexes in the virus genome.

## Results

**Figure 1:**
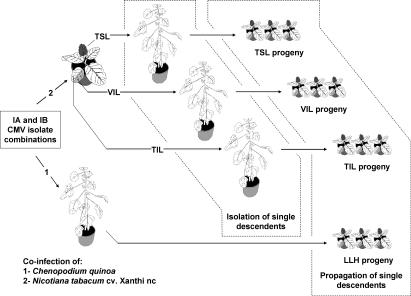
Outline of Double Inoculation Experiments with IA and IB CMV Isolates Four IA and IB isolate combinations were inoculated in C. quinoa or in tobacco cv. Xanthi-nc. In tobacco, total RNA was extracted from inoculated (TIL) and systemic infected (TSL) leaves and encapsidated RNA was extracted from inoculated leaves (VIL). Local-lesion descendents were isolated in C. quinoa and transferred to tobacco Xanthi-nc for propagation.

The frequency of reassortants and recombinants in progenies from double inoculations with CMV isolates belonging to subgroups IA (genetic type AAA.A, i.e., allele A at loci *i, j, k*_1_, and *k*_2_, ORFs 1a, 2a, 3a, and CP, respectively; see Materials and Methods) and IB (genetic type BBB.B, i.e., allele B at the four loci) was analyzed in *Chenopodium quinoa,* a local-lesion host for CMV, and in tobacco, a host in which CMV infection is systemic. In a local-lesion host, infection is limited to its initial steps, as virus replication and movement are restricted to few cells around those initially infected. These local lesions are the equivalent for plant viruses of lysis plaques for lytic viruses, and, similarly, provide a means for their biological cloning. Thus, each of the resulting local lesions obtained in C. quinoa represent a single descendent from the mixed infections. Frequency of descendents after mixed inoculation of the local-lesion host C. quinoa (here named LLH) should primarily reflect their relative infectivity. In tobacco, the progeny of the mixed infections was analyzed at three moments of the systemic colonization of the plant, so that progeny composition could be modulated by different processes in the virus life cycle. For this, three different RNA preparations were used. First, total RNA extracts were obtained from the inoculated leaves (named TIL), in which infection initiation, replication, and cell-to-cell colonization of parenchyma cells had occurred; second, encapsidated RNA was extracted after purification of viral particles from the same inoculated leaves (named VIL), so that a further step in the virus life cycle, i.e., encapsidation, had occurred; third, total RNA was extracted from upper leaves (named TSL), after long-distance movement in the phloem and colonization of new leaves had occurred. RNA preparations from tobacco were inoculated on C. quinoa leaves to obtain single local-lesion descendents. Local lesions from mixed-infection progenies in both hosts were individually transferred to young Xanthi-nc. tobacco plants for multiplication, and their genotype was characterized for allelic values A or B in each of the four analyzed loci (see Materials and Methods). The experimental procedure (see Materials and Methods) is summarized in Figure 1.

Double inoculations were done with four different pair combinations of IA and IB isolates in four parallel experiments (see Materials and Methods). The infectivity of each of the eight isolates was estimated by single-lesion assay on *C. quinoa,* after linear regression of the mean number of lesions per half leaf against the logarithmic transformation of RNA concentration in the inoculum (unpublished data). The comparison of the slope and intercept of these regressions showed that the infectivity of subgroup IA and IB isolates within each pair combination did not differ significantly (0.15 < *p* < 0.98 for the different pairs). Thus, in double inoculations of each isolate pair in C. quinoa or in tobacco, the initial frequency ratio between the type IA and type IB genomic RNAs, i.e., between alleles A and B at loci *i* in RNA1, *j* in RNA2, and *k*_1_.*k*_2_ in RNA3, was 0.5:0.5.

### Genetic Exchange during Infection of LLH

Frequency distributions of parental, reassortant, and recombinant genotypes in LLH progenies of the four analyzed isolate combinations (see Table S1) were homogeneous (i.e., did not differ at 95% level of confidence) between combinations I, III, and IV, or II, III, and IV. Genotype frequencies pooled over the four isolate combination progenies are presented in Table 1 (LLH pooled progeny), which also indicate the frequency of alleles A and B at loci *i, j,* and *k*_1_.*k*_2_. Because the relative proportion of alleles A and B at each loci in the mixed inoculum, measured in terms of infectivity, was 0.5:0.5, the expected frequency of each genotype under the hypothesis of random reassortment can be calculated from the combinatorial probability of these allelic proportions at each of the three loci, assuming independence (linkage equilibrium) in the distribution of the three genomic segments: 0.5 × 0.5 × 0.5 = 0.125. Each of the parental and the six possible reassortant genotypes occurred in LLH progenies, but with large differences in frequency, so that in no case the genotype frequency distribution did fit that expected from random (0.125) under the linkage equilibrium hypothesis (*p* < 0.0001 for every progeny). Genotypes with allele A at loci *k*_1_.*k*_2_ represented 0.78 of the pooled progeny: genotypes AAA.A, BAA.A, and BBA.A were the most frequent ones, the frequency of ABA.A and of genotypes with allele B at loci *k*_1_.*k*_2_ was always lower and similar.

**Table 1:**
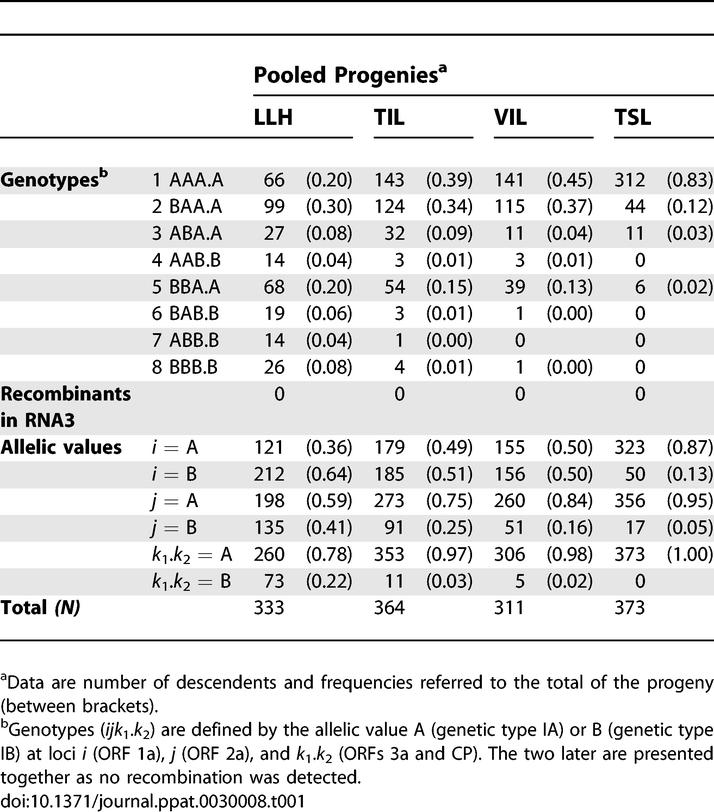
Frequency Distribution of Genetic Types in Pooled Progenies from Double Inoculations of IA and IB CMV Isolates

Genotype frequency distributions were analyzed by fitting log-linear models, which express the overall logarithmic-deviation of observed genotype frequencies (once fitted by the model) from those expected under the null hypothesis (see Materials and Methods). Parameters α*_i_,* β*_j_,* and γ*_k_* in the model (Table 2) represent the positive or negative log-deviations from the random expected genotype frequency (0.125) that are due to changes in frequency of alleles A or B at loci *i* (α*_i_* ), *j* (β*_j_*), and *k*_1_.*k*_2_ (γ*_k_*) (*i, j, k*_1_, and *k*_2_ = A, B). Parameters αβ*_ij_,* αγ*_ik_,* βγ*_jk_,* and αβγ*_ijk_* represent additional log-deviations due to associations among loci (i.e., due to linkage disequilibrium) from the linkage equilibrium expectation under the observed allele frequencies. For example, in the case of LLH pooled progeny, γ_A_ and γ_B_ (Table 2) indicate, respectively, increase (γ_A _> 0) and decrease (γ_B _< 0) factors of e^γA^ and e^γB^ in frequencies of allele A and B at loci *k*_1_.*k*_2_ from their random expectation of 0.5. The overall effect due to the change in A:B proportion at this loci is expressed by a unique parameter γ*_k_* = 0.63511, calculated as half the difference γ_A_ − γ_B_, which indicates an increase (γ*_k_* > 0) factor of e^2γ*k*^ in frequency of allele A over frequency of allele B. Parameters of the model for the LLH pooled progeny (Table 2) showed a significant increase in frequency of allele A relative to allele B at loci *k*_1_.*k*_2_ (*p* < 0.0001) and at locus *j* (β*_j_* = 0.19149, *p* = 0.0005), while allele A decreased in frequency relative to allele B at locus *i* (α*_i_* = −0.28039, *p* < 0.0001). Models also indicated significant (*p* = 0.007) homologous association between locus *j* in RNA2 and loci *k*_1_.*k*_2_ in RNA3, resulting in increased genotype frequency of pairs *i*AA.A and *i*BB.B and decreased frequency of pairs *i*AB.B and *i*BA.A (*i* = A, B) by factors of e^βγAA^, e^βγBB^, e^βγAB^, and e^βγBA^ (Table 2) from their linkage equilibrium expectation given the observed allele frequencies. The overall effect of this association, βγ*_jk_* = 0.18611, calculated as the half mean of βγ_AA_ − βγ_AB_ and βγ_BB_ − βγ_BA_, represents an increase factor (βγ*_jk_* > 0) of e^2βγij^ in the frequency of homologous over heterologous allele combinations at loci *j* and *k*_1_.*k*_2_. A standard measure of linkage disequilibrium is *Q,* which is directly calculated from βγ*_jk_* (see Materials and Methods) and takes the value 0.3559. Another common standard measure of linkage disequilibrium is *D′,* which in the case of those two loci was *D′* = 0.2397 (see also Materials and Methods). Significance of model parameters for each isolate combination is indicated in Table S1.

**Table 2:**
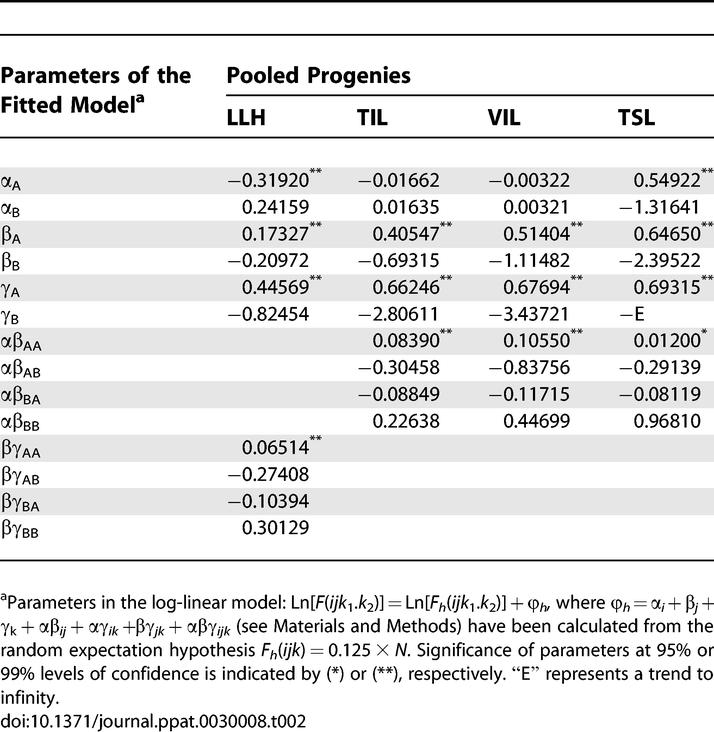
Analysis of Frequency Distribution of Genetic Types in Pooled Progenies from Double Inoculations of IA and IB CMV Isolates and Fitting of Log-Linear Models

No recombinants at RNA3 were found in the progeny of any of the four combinations of IA and IB isolates (Tables 1 and S1).

### Genetic Exchange during Infection of a Systemic Host

**Figure 2:**
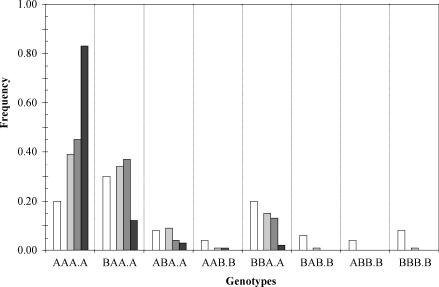
Variation of Genotype Frequencies in Pooled Progenies from Double Inoculations of IA and IB CMV Isolates Frequencies in TIL (black box), VIL (dark-gray box), and TSL (light-gray box) progenies are compared to those of LLH (white box) progenies. doi:10.1371/journal.ppat 0030008.g002

Frequency of genotype distributions of TIL, VIL, and TSL progenies for all four isolate combinations are detailed in Tables S2, S3, and S4, respectively, and indicated in Table 1 for the corresponding TIL, VIL, and TSL pooled progenies. Homogeneity of frequency distribution of genotypes was found for combinations I and II in TIL, for combinations II and IV in VIL, and for all four isolate combinations in TSL (*p* = 0.054), being highly homogeneous for combinations I, II, and IV in TSL (*p* = 0.978). In all progenies from the systemic host, genotypes AAA.A, BAA.A, and BBA.A were the most frequent, genotypes with allele B at loci *k*_1_.*k*_2_ being at low frequency (TIL and VIL), or not detected at all (TSL). Frequency of genotypes AAA.A and BAA.A together made 0.73 of the total population in TIL and progressively increased to 0.82 in VIL and to 0.95 in TSL (Table 1). Figure 2 shows the variation of all genotype frequencies in the pooled progenies from TIL to VIL and TSL, as compared to LLH.

Analysis of genotype frequency distributions in TIL, VIL, and TSL pooled progenies by log-linear models showed a significant increase of A:B allele proportions from their random expectation hypothesis (0.5:0.5) at loci *j* and *k*_1_.*k*_2_ in TIL and VIL, or at the three loci *i, j, k*_1_.*k*_2_ in TSL, as shown by model parameters (Table 2). A significant homologous association between loci *i* and *j* was detected in the three pooled progenies: αβ*_ij_* = 0.17584, *Q* = 0.3379, *D′* = 0.2627 (*p* = 0.0053) for TIL; αβ*_ij_* = 0.37680, *Q* = 0.6373, *D′* = 0.5672 (*p* < 0.0001) for VIL; and αβ*_ij_* = 0.33817, *Q* = 0.5891, *D′* = 0.2528 (*p* = 0.0166) for TSL.

Variation of genotype frequency distributions, as systemic colonization of the host progressed could also be analyzed by log-linear models, estimating the deviations of genotype frequencies in a progeny from the expected frequencies resulting from allelic frequencies observed in the previous step in the virus life cycle, assuming a linkage equilibrium distribution. For example, deviations in TIL pooled progeny distribution were estimated from linkage equilibrium distribution of allelic frequencies in the LLH pooled progeny, taking it as the null hypothesis in the model. Parameters of the model could be easily computed as the difference between that in TIL and that in LLH, as estimated in both cases from the random expectation (Table 2). It was found that deviations of TIL from LLH expectation were significant at the three loci, indicating the increase of the relative frequency of allele A: α*_i_* = 0.26391, β*_j_* = 0.35781, γ*_k_* = 1.09918 (*p* < 0.0001). Deviations of VIL progeny from TIL expectation were only significant at locus *j:* β*_j_* = 0.35781 (*p* = 0.0002). TSL pooled progeny was analyzed from both TIL and VIL expectation. Deviations of TSL from both TIL and VIL expectation were significant at loci *i* and *j* [α*_i_* = 0.94929, β*_j_* = 0.97155 (*p* < 0.0001) for TIL; α*_i_* = 0.93603, β*_j_* = 0.70643 (*p* < 0.0001) for VIL].

No recombinants at RNA3 were detected in any TIL, VIL, or TSL progeny (Tables 1 and S2–S4).

## Discussion

Research during the last two decades has shown the important role of genetic exchange by reassortment of genomic segments, or by recombination, in RNA virus evolution [2,3,16,17,23]. However, although there are reports of virus populations at linkage equilibrium [24], data from numerous systems indicate that the frequency of reassortant genotypes in natural populations of viruses with segmented genomes widely departs from random expectations, and, similarly, frequency of recombinants is lower than in experiments in the culture plate or in the greenhouse [22,25,26]. In fact, evidence suggests that selection against heterologous gene combinations occurs, perhaps due to coadaptation between the genes within a viral genome [21,27–30]. Little is known about the factors that may operate along the virus life cycle affecting the final frequency of reassortant and recombinant genotypes and its epidemiological impact in nature [17]. Here, we address these questions. The approach was to estimate the frequency of reassortants and of recombinants at RNA3, in descents of double inoculations with CMV isolates belonging to subgroup IA and subgroup IB, which differ by about 12% in nucleotide sequence [18]. This level of genetic divergence should not hinder the possibility of genetic exchange, as CMV isolates in subgroups IA, IB, and II are known to produce viable reassortants and recombinants, which under experimental conditions are infectious and will accumulate to similar levels as the parental strains in single infection [18]. Also, a recent report has demonstrated an extremely high frequency of reassortants in natural populations of highly diverged (up to 50%) viruses within the *Cystoviridae* family [24]. The experiments were done with four pairs of CMV isolates, and analyses of progenies were planned to identify constraints to genetic exchange at four steps in the virus life cycle: (i) infection of leaf cells, (ii) colonization of the inoculated leaf, which involves infection of leaf cells and cell-to-cell movement, (iii) encapsidation of viral RNA, and (iv) colonization of upper, noninoculated leaves, which involves systemic movement through the phloem. Step (i) was analyzed in a local-lesion host, *C. quinoa,* through the estimation of genotype frequencies in the resulting local-lesion populations (populations LLH), and steps (ii–iv) were analyzed in a systemic host, tobacco, in progeny populations represented by total RNA preparations from inoculated or systemically infected leaves (TIL and TSL, respectively) or by virion RNA from inoculated leaves (VIL).

Results from our experiments confirm that coinfection with IA and IB CMV isolates in the four combinations assayed resulted in the production of viable reassortants in the six possible combinations (Table 1). However, the frequency distributions of the eight parental and reassortant genotypes departed largely from random even at the very beginning of the infection process, as shown by data from LLH progenies (Tables 1 and S1). Different genotype frequencies could reflect differences in the relative ability of genomic segments carrying alleles A and B to infect cells, or alternatively, differences in the capacity to infect the host plant among the genotypes, i.e., differences in their relative fitness. Local-lesion assays had shown that infectivity of the parental isolates in each combination was equivalent. Hence, results from LLH progenies indicate differences in fitness among genotypes for the formation of a single local lesion, i.e., the establishment of a successful infection at an initial cell, plus restricted virus movement to a few surrounding cells. Deviations from the null hypothesis could also be due to sampling bias during the isolation-infection processes, e.g., selection against particular genotypes during amplification of single-lesion descents in tobacco, but this possibility most probably can be discarded, given the high number of lesions transferred and the high success of infection in tobacco from necrotic local lesions (about 80%). Also, random genetic drift associated with sampling or with population bottlenecks during local-lesion cloning cannot be discarded, but, again, the high number of lesions transferred for each progeny should minimize the impact of drift in the obtained results. Hence, as far as departures from expectation can be attributed to selection, the quantity *a_h_*(*ijk*_1_.*k*_2_) = −1 + *e* ^ϕ^ (see Materials and Methods) represents a coefficient of selection for each genotype, which can be calculated from parameters of the log-linear models. Differences in coefficients of selection among the different genotypes were even higher in the systemic host than in the local-lesion host, and increased as the infection process progressed, i.e., in progeny populations TIL, VIL and TSL. Biological cloning of descents in C. quinoa should introduce a bias in genotype frequencies, as not all genotypes were equally infectious to this host, but the effect would be the same for populations TIL, VIL, and TSL. Thus, each step during colonization of the systemic host resulted in stronger genotype selection, so that genotype frequency distribution departed more and more from random, and only three genotypes were detected in the four TSL progenies. It is to be noted that differences of genotype frequency distribution among the progenies of the four analyzed isolate combinations occurred in TIL and VIL, but frequency distribution was homogeneous for the four TSL progenies, indicating that similar selective pressures were operating in each progeny regardless of the nature of the parental isolates.

Nonrandom distribution of reassortants in experimental populations has been reported for other RNA viruses with segmented genomes and has been interpreted as due to specific associations between genomic segments related to functional interactions between the RNAs or their protein products [27,30,31]. Alternatively, nonrandom reassortment has been explained as due to selective advantages of specific genome segments [29,32–34]. Because differences in selective advantage between genomic segments will not be independent of genetic context, both interpretations share a common basis and relate to the concept of coadaptation of gene complexes within the viral genome [35]. Occurrence of epistasis and coadapted gene complexes in genomes has important consequences for the evolution of natural populations [35], and efforts have been made to estimate epistasis on viral genomes, mostly based on analysis of fitness effects of two or more point mutations [36,37], rather than on the fitness of hybrids in progenies from crosses. Our data indicate an advantage of allele A over allele B at loci *k*_1_.*k*_2_ in RNA3 since the earlier stages of infection (populations LLH and TIL, Table 1), so that in systemically infected leaves (TSL populations) genotypes with allele B at loci *k*_1_.*k*_2_ were not detected (Table 1). This was also the case for locus *j* in RNA2, the advantage of allele A, since the earlier stages of infection were particularly noticeable after encapsidation. Only at locus *i* in RNA 1 was allele B not at disadvantage relative to allele A in the inoculated leaves, but allele B was at disadvantage in systemically infected leaves, where the parental genotype AAA.A prevailed in the four progenies (Table 1). Selection against particular alleles at the analyzed loci was not independent of their genetic background, as significant associations of homologous gene combinations, indicating linkage disequilibrium, were found for some loci *(j* and *k_1_.k_2_* or *i* and *j)* in all pooled progenies. Hence, our data support the hypothesis of coadaptation of the four analyzed genes within the CMV genome, and show the higher fitness of genotype AAA.A in the assayed host and conditions. This result is in agreement with a recent report on the diminished competitive ability of a CMV reassortant [38]. The results of the present work also agree with analyses of the genetic structure of field populations of CMV, where isolates from subgroups IA and IB were at similar frequencies and were often coinfecting the same plant, but selection against reassortants and most recombinants (i.e., against heterologous gene combinations) seemed to occur [21,22].

Mechanisms for nonrandom association of genomic segments have been proposed for other viruses, with selective advantages for specific genome segments being functionally linked to differences in replication efficiency [32,33], infectivity after assembly into particles [34], or interaction with host cell factors [29]. For CMV, allele A at loci *k*_1_.*k*_2_ in RNA3 could have an advantage relative to allele B in competition for infection sites, replication, or cell-to-cell movement. RNA3 encodes promoters and other regulatory sequences for its replication as well as the two proteins, 3a and CP, required for cell-to-cell movement [18]. The relative increase in frequency of allele A relative to allele B at locus *j* in RNA2 as cell-to-cell movement progresses (compare data for LLH and TIL) or during encapsidation (compare data for TIL and VIL), suggests that the homologous combination of RNA2 and RNA3 performs better than the heterologous cell-to-cell movement and encapsidation functions. This would not be the case for the combination of RNA1 and RNA3. There was a sharp increase in relative frequency of allele A at locus *i* in RNA1 associated to systemic movement, suggesting that the homologous combination of the three RNAs performs the function of systemic colonization better, which occurs in the form of assembled viral particles and may depend on interactions between the CMV capsid and host factors [39,40]. Alternatively, a higher fitness for homologous allele combinations in RNAs 1 and 2, related to the interaction of their protein products in the viral replicase [41], could lead to a delayed increase of allele A at locus *i* as it increases at locus *j,* and explain the association between loci *i* and *j* observed in the systemic host.

We have also analyzed the frequency of RNA3 recombinants between loci *k*_1_ and *k*_2_ in the progenies of the four IA and IB CMV isolate combinations. No recombinant genotype was detected among 1,381 descendents in LLH, TIL, VIL, or TSL progenies. Thus, the probability of finding one recombinant RNA3 was lower than 0.004, at a 95% confidence level, assuming that this probability was the same for the 16 progenies. Otherwise, the probability would be even lower and the 0.004 value would be an upper threshold estimate. The observed differences in fitness between type A and B RNA3, since the earlier stages of infection, might affect the probability of coinfection in the same cell, and, thus, the probability of recombination, as reported for CMV and *Tomato aspermy virus* [42,43]. In addition, exclusion of different CMV strains from infected cells [44] would also decrease the probability of recombination.

In the progeny populations analyzed in this work, heterologous allele combinations were underrepresented relative to their expectation under the null hypothesis of linkage equilibrium. This shows the existence of constraints to genetic exchange linked to the various steps of host infections and colonization. Our results show that, whatever the reason for an initial disadvantage of genotypes with heterologous gene combinations, selection against these genotypes becomes stronger as host colonization proceeds so that the fittest genotypes would be the most available for host-to-host transmission and the heterologous gene combinations would disappear from the population. These results are important for understanding the role of genetic exchange in virus evolution and may be relevant for applied aspects of plant virology, as they might affect the durability of resistance genes [45], or the ecological risks of virus-resistant transgenic plants [46]. In a more general context, these results support the hypothesis of coadaptation of gene complexes within a genome, which might be particularly relevant for the small, compacted, nonredundant genomes of RNA viruses.

## Materials and Methods

### Virus isolates.

Eight CMV isolates were used in this work, four belonging to subgroup IA and four to subgroup IB. These isolates were derived from field-infected zucchini squash or tomato plants sampled in Spain between 1992 and 1994, when both types of isolates were frequent in the field [21] and were characterized as belonging to subgroups IA or IB by ribonuclease protection assay as described [21]. Isolates were multiplied in Nicotiana tabacum cv. Xanthi-nc, virion stocks were purified from systemically infected leaves as in [47], and virion RNA was extracted with phenol and sodium dodecyl sulfate.

### Generation of progenies for the analysis of genetic exchange.

Genetic exchange was analyzed in progenies from double inoculations with four different pair combinations of one isolate belonging to subgroup IA and one isolate belonging to subgroup IB. Coinoculations were replicated in ten half-leaves of the local-lesion host *Chenopodium quinoa,* and in five plants of the systemic host Nicotiana tabacum cv. Xanthi-nc. All inoculations were with virion RNA in 0.1 M Na_2_HPO_4_, in leaves previously dusted with carborundum. RNA concentration of each isolate in the inoculum was such that the isolate's infectivity ratio was 0.5:0.5. The relative infectivity of the IA and IB isolates in each combination was estimated by local-lesion assays in ten half-leaves at three different inoculum concentrations in the range 0.1–2.5 μg RNA/ml.

For the tobacco plants, total RNA and virion-encapsidated RNA was purified from inoculated leaves 7 d post inoculation (dpi), and total RNA was purified from systemically infected leaves 12 dpi. Total RNA was extracted from 200 mg of plant tissue as in [48]. Virus particles and virion RNA were purified as in [49] from inoculated leaves. In this way, three different RNA preparations were obtained from each infected plant, representing different progeny populations: total RNA from inoculated leaves (TIL), virion RNA from inoculated leaves (VIL), and total RNA from systemically infected leaves (TSL). Total or virion RNA from each of the five infected plants per treatment was pooled, diluted at a ratio of 50 mg tissue/ml, and inoculated onto half-leaves of *C. quinoa,* for the cloning of single-lesion descendents. About 150 local lesions per progeny were individually transferred to small Xanthi-nc tobacco plants for multiplication, and 15 d later total RNA was extracted from these tobacco plants for the genetic characterization of descendents.

### Genetic characterization of single-lesion descendents.

Four pairs of oligonucleotide probes specific for ORFs encoding proteins 1a, 2a, 3a, and CP of CMV isolates in subgroups IA and IB were designed on the basis of nucleotide sequence information from ten CMV isolates of subgroup IA and eight CMV isolates of subgroup IB [21,50] (unpublished data): the first pair, CMV1A (5′CATTAATGTCTATTCG3′) and CMV1B (5′CGTTGATGTCGATACG3′) were complementary to positions 1,330–1,346 of CMV RNA1; the second pair, CMV2A (5′GCGCTGTGAATAACGG3′) and CMV2B (5′GCGCAGTAAACAACGG3′) were complementary to positions 1,506–1,521 of CMV RNA2; the third pair, CMV3a-A (5′GACCCTTCAGCATCAG3′) and CMV3a-B (5′GATCCCTCAGCGTCGG3′) were complementary to positions 421–436 of CMV RNA3; the fourth pair, CMVCP-A (5′GGACTCCAGATGCGGC3′) and CMVCP-B (5′GAACGCCGGATGCAGC3′) were complementary to positions 1,722–1,737 of CMV RNA3. Dot blot hybridization with these eight oligonucleotide probes, 5′-labeled with ^32^P [51], unequivocally identified genetic types IA and IB in the four analyzed ORFs for the eight IA and IB parental CMV isolates (unpublished data).

### Statistical analysis.

Frequency distributions of genotypes in progenies were compared to expected frequency distributions according to the null hypothesis being tested, which was derived from the frequencies of IA and IB genetic types at each analyzed CMV ORF: either 0.5:0.5 at the inoculum, or as resulted from genotype distributions in previous steps of the virus life cycle, always assuming linkage equilibrium. Comparison between observed and expected frequencies was performed by the chi-square (*χ* ^2^) goodness of fit test. Comparison of frequency distributions of genotypes for the four CMV isolate combinations was done by the log-likelihood ratio test *(G)* for homogeneity of replicates tested for goodness of fit [52]. Genotype distributions were analyzed for independence among genomic segments (linkage equilibrium) by three-way contingency tables, which were solved upon the use of log-linear models [52]. These models were adapted to take the form: Ln[*F*(*ijk*_1_.*k*_2_)] = Ln[*F_h_*(*ijk*_1_.*k*_2_)] + ϕ*_h_,* where *F*(*ijk*_1_.*k*_2_) is the model estimate for the observed frequency of genotype *ijk*_1_.*k*_2_ (*i, j, k*_1_, and *k*_2_ are loci 1a, 2a, 3a, and CP, respectively, and may take the allelic values A for genetic type IA and B for genetic type IB); *F_h_*(*ijk*_1_.*k*_2_) is the expected frequency of that genotype under the null hypothesis *h,* and ϕ*_h_* is the overall log-frequency deviation under that hypothesis, where ϕ*_h_* = α*_i_* + β*_j_* + γ*_k_* + αβ*_ij_* + αγ*_ik_* + βγ*_jk_* + αβγ*_ijk_ ;* α*_i_,* β*_j_,* γ*_k_* are log-deviations due to the frequencies of alleles A or B at loci *i, j,* and *k*_1_.*k*_2_ and αβ*_ij_,* αγ*_ik_,* βγ*_jk,_* and αβγ*_ijk_* are the log-deviations due to associations among loci in different genomic segments: *i, j,* and *k*_1_.*k*_2_. Deviation parameters were computed by fitting the model to observed and expected frequencies for each genotype [52]. When significant association among genomic segments was found, linkage disequilibrium was measured by the two standard metrics *Q* and *D′* [53]: *Q* may be directly computed from association parameters in the model as *Q* = (λ−1) / (λ+1), where λ = e^4·A^, A being the model parameter; *D′* measures linkage disequilibrium relative to its maximum value under the observed allele frequencies, *D′* = *D*/*D*_max_ (for a set of two biallelic loci in which alleles A and B have frequencies *p*_A+_ and *p*_B+_ at the first locus, *p*_+A_ and *p*_+B_ at the second locus, and *p*_AA_, *p*_AB_, *p*_BA_, *p*_BB_ are frequencies of the four possible genotype combinations, then *D* = *p*_AA_ *p*_BB_ – *p*_AB_ *p*_BA_, and *D*_max_ is the lesser of *p*_A+_ *p*_+B_ and *p*_+A_ *p*_B+_ if *D* is positive, or the lesser of *p*_A+_ *p*_+A_ and *p*_B+_ *p*_+B_ if *D* is negative [53]). The quotient *F*(*ijk*_1_.*k*_2_)/*F_h_*(*ijk*_1_.*k*_2_) = 1 + *a_h_*(*ijk*_1_.*k*_2_) represents the relative departure from expectation hypothesis *h.* Its deviation from one would be a coefficient of selection in case of fitness variation, which can be computed as *a_h_*(*ijk*_1_.*k*_2_) = −1 + *e* ^ϕ^. For all statistical tests, the probability of rejecting the null hypothesis was calculated by *χ* ^2^ or *G* exact methods, or by Monte Carlo simulations with 10^6^ replicates, using the SAS Statistical v 9.1 package (SAS Institute, http://www.sas.com).

## Supporting Information

Table S1 Frequency Distribution of Genetic Types in Progenies from Double Inoculations of IA and IB CMV Isolates on the LLH Chenopodium quinoa
Data are number of descendants and frequencies referred to the total of the progeny (between brackets). Genotype distributions with the same letter did not differ at a 95% level of confidence.
(72 KB DOC)

Table S2 Frequency Distribution of Genetic Types in Progenies from Double Inoculations of IA and IB CMV Isolates on the Systemic Host Nicotiana tabacum cv. Xanthi-nc Progenies recovered from total RNA extracts of TIL. Data are number of descendants and frequencies referred to the total of the progeny (between brackets). Genotype distribution with the same letter did not differ at a 95% level of confidence.
(65 KB DOC)

Table S3 Frequency Distribution of Genetic Types in Progenies from Double Inoculations of IA and IB CMV Isolates on the Systemic Host Nicotiana tabacum cv. Xanthi-nc Progenies recovered from virion-encapsidated RNA from VIL. Data are number of descendants and frequencies referred to the total of the progeny (between brackets). Genotype distribution with the same letter did not differ at a 95% level of confidence.
(71 KB DOC)

Table S4 Frequency Distribution of Genetic Types in Progenies from Double Inoculations of IA and IB CMV Isolates on the Systemic Host Nicotiana tabacum cv. Xanthi-nc Progenies recovered from total RNA extracts of TSL. Data are number of descendants and frequencies referred to the total of the progeny (between brackets). Genotype distributions with the same letter did not differ at a 95% level of confidence.
(65 KB DOC)

### Accession Numbers

The GenBank (http://www.ncbi.nlm.nih.gov/Genbank) accession numbers for the nucleotide positions in the sequences for CMV discussed in this paper are RNA1 (D00356), RNA2 (D00355), and RNA3 (D10538).

## Abbreviations

CMV: Cucumber mosaic virus, CP, coat protein
LLH: local-lesion host
ORF: open reading frame
TIL: total RNA extracted from inoculated leaves
TSL: total RNA extracted from systemically infected leaves
VIL: encapsidated RNA extracted from inoculated leaves

## References

[ppat-0030008-b001] Chao L (1992). Evolution of sex in RNA viruses. Trends Ecol Evol.

[ppat-0030008-b002] Guan Y, Poon LLM, Cheung CY, Ellis TM, Lim W (2004). H5N1 influenza: A protean pandemic threat. Proc Natl Acad Sci U S A.

[ppat-0030008-b003] Reid AH, Taubenberger JK (2003). The origin of the 1918 pandemic influenza virus: A continuing enigma. J Gen Virol.

[ppat-0030008-b004] Russeell CJ, Webster RG (2005). The genesis of a pandemic influenza virus. Cell.

[ppat-0030008-b005] Gibbs MJ, Armstrong JS, Gibbs AJ (2001). Recombination in the hemagglutinin gene of the 1918 “Spanish flu.”. Science.

[ppat-0030008-b006] Javier RT, Sedarati F, Stevens JG (1986). Two avirulent herpes-simplex viruses generate lethal recombinants in vivo. Science.

[ppat-0030008-b007] Gibbs MJ, Weiller GF (1999). Evidence that a plant virus switched hosts to infect a vertebrate and then recombined with a vertebrate-infecting virus. Proc Natl Acad Sci U S A.

[ppat-0030008-b008] Hu WS, Rhodes T, Dang Q, Pathak V (2003). Retroviral recombination: Review of genetic analyses. Front Biosci.

[ppat-0030008-b009] Rest JS, Mindell DP (2003). Retroids in Archaea: Phylogeny and lateral origins. Mol Biol Evol.

[ppat-0030008-b010] Legg JP, Thresh JM (2000). *Cassava mosaic virus* disease in East Africa: A dynamic disease in a changing environment. Virus Res.

[ppat-0030008-b011] Monci F, Sánchez-Campos S, Navas-Castillo J, Moriones E (2002). A natural recombinant between the geminiviruses Tomato yellow leaf curl Sardinia virus and Tomato yellow leaf curl virus exhibits a novel pathogenic phenotype and is becoming prevalent in Spanish populations. Virology.

[ppat-0030008-b012] Müller HJ (1964). The relation of recombination to mutational advance. Mutat Res.

[ppat-0030008-b013] Chao L, Tran TT, Tran TT (1997). The advantage of sex in the RNA virus phi6. Genetics.

[ppat-0030008-b014] Chao L, Tran T, Matthews C (1992). Müller's ratchet and the advantage of sex in the RNA virus phi6. Evolution.

[ppat-0030008-b015] Froissart R, Roze D, Uzest M, Galibert L, Blanc S (2005). Recombination every day: Abundant recombination in a virus during a single multi-cellular host infection. PLoS Biol.

[ppat-0030008-b016] García-Arenal F, Fraile A, Malpica JM (2001). Variability and genetic structure of plant virus populations. Annu Rev Phytopathol.

[ppat-0030008-b017] Worobey M, Holmes EC (1999). Evolutionary aspects of recombination in RNA viruses. J Gen Virol.

[ppat-0030008-b018] Palukaitis P, García-Arenal F (2003). Cucumoviruses. Adv Virus Res.

[ppat-0030008-b019] Roossinck MJ, Zhang L, Hellwald K (1999). Rearrangements in the 5′ nontranslated region and phylogenetic analyses of *Cucumber mosaic virus* RNA3 indicate radial evolution of three subgroups. J Virol.

[ppat-0030008-b020] Roossinck MJ (2002). Evolutionary history of *Cucumber mosaic virus* deduced by phylogenetic analyses. J Virol.

[ppat-0030008-b021] Fraile A, Alonso-Prados JL, Aranda MA, Bernal JJ, Malpica JM (1997). Genetic exchange by recombination or reassortment is infrequent in natural populations of a tripartite RNA plant virus. J Virol.

[ppat-0030008-b022] Bonnet J, Fraile A, Sacristán S, Malpica JM, García-Arenal F (2005). Role of recombination in the evolution of natural populations of *Cucumber mosaic virus,* a tripartite RNA plant virus. Virology.

[ppat-0030008-b023] Turner PE (2003). Searching for the advantages of virus sex. Orig Life Evol Biosph.

[ppat-0030008-b024] Silander OK, Weinreich DM, Wright KM, O'Keefe KJ, Rang CU (2005). Widespread genetic exchange among terrestrial bacteriophages. Proc Natl Acad Sci U S A.

[ppat-0030008-b025] Henderson WW, Monroe MC, Jeor SCS, Thayer WP, Rowe JE (1995). Naturally occurring Sin Nombre virus genetic reassortants. Virology.

[ppat-0030008-b026] Palombo EA, Bugg HC, Masendycz PJ, Coulson BS, Barnes GL (1996). Multiple-gene rotavirus reassortants responsible for an outbreak of gastroenteritis in central and northern Australia. J Gen Virol.

[ppat-0030008-b027] Nibert ML, Margraf RL, Coombs KM (1996). Nonrandom segregation of parental alleles in reovirus reassortants. J Virol.

[ppat-0030008-b028] Perry KL, Francki RIB (1992). Insect-mediated transmission of mixed and reassorted cucumovirus genomic RNAs. J Gen Virol.

[ppat-0030008-b029] Graham A, Kudesia G, Allen AM, Desselberger U (1987). Reassortment of human rotavirus possessing genome rearrangements with bovine rotavirus: Evidence for host-cell selection. J Gen Virol.

[ppat-0030008-b030] Hanada K, Harrison BD (1977). Effects of virus genotype and temperature on seed transmission of nepoviruses. Ann Appl Biol.

[ppat-0030008-b031] Lubeck MD, Palese P, Schulman JL (1979). Nonrandom association of parental genes in influenza A virus recombinants. Virology.

[ppat-0030008-b032] Qiu WP, Geske SM, Hickey CM, Moyer JW (1998). Tomato spotted wilt Tospovirus genome reassortment and genome segment-specific adaptation. Virology.

[ppat-0030008-b033] Urquidi V, Bishop DHL (1992). Nonrandom reassortment between the tripartite RNA genomes of La Crosse and snowshoe hare viruses. J Gen Virol.

[ppat-0030008-b034] Ward RL, Knowlton DR, Hurst PFL (1988). Reassortant formation and selection following coinfection of cultured cells with subgroup-2 human rotaviruses. J Gen Virol.

[ppat-0030008-b035] Fenster CB, Galloway LF, Chao L (1997). Epistasis and its consequences for the evolution of natural populations. Trends Ecol Evol.

[ppat-0030008-b036] Burch CL, Chao L (2000). Evolvability of an RNA virus is determined by its mutational neighborhood. Nature.

[ppat-0030008-b037] Sanjuán R, Moya A, Elena SF (2004). The contribution of epistasis to the architecture of fitness in an RNA virus. Proc Natl Acad Sci U S A.

[ppat-0030008-b038] Takeshita M, Kikuhara K, Kuwata S, Furuya N, Takanami Y (2004). Competition between wild-type virus and a reassortant from subgroups I and II of CMV and activation of antiviral responses in cowpea. Arch Virol.

[ppat-0030008-b039] Blackman LM, Boevink P, Cruz SS, Palukaitis P, Oparka KJ (1998). The movement protein of *Cucumber mosaic virus* traffics into sieve elements in minor veins of Nicotiana clevelandii. Plant Cell.

[ppat-0030008-b040] Requena A, Simón-Buela L, Salcedo G, García Arenal F (2006). Potential involvement of a cucumber homolog of phloem protein 1 in the long-distance movement of *Cucumber mosaic virus* particles. Mol Plant Microbe Interact.

[ppat-0030008-b041] Hayes RJ, Buck KW (1990). Complete replication of a eukaryotic virus RNA in vitro by a purified RNA-dependent RNA polymerase. Cell.

[ppat-0030008-b042] Aaziz R, Tepfer M (1999). Recombination between genomic RNAs of two cucumoviruses under conditions of minimal selection pressure. Virology.

[ppat-0030008-b043] Sackey ST, Francki RIB (1990). Interaction of cucumoviruses in plants: Persistance of mixed infections of cucumber mosaic and tomato aspermy viruses. Physiol Mol Plant Pathol.

[ppat-0030008-b044] Takeshita M, Shigemune N, Kikuhara K, Furuya N, Takanami Y (2004). Spatial analysis for exclusive interactions between subgroups I and II of *Cucumber mosaic virus* in cowpea. Virology.

[ppat-0030008-b045] García-Arenal F, McDonald BA (2003). An analysis of the durability of resistance to plant viruses. Phytopathology.

[ppat-0030008-b046] Tepfer M (2002). Risk assessment of virus-resistant transgenic plants. Annu Rev Phytopathol.

[ppat-0030008-b047] Lot H, Marrou J, Quiot JB, Esvan C (1972). Contribution à l'étude du virus de la mosaïque du concombre (CMV). Méthode de purification rapide du virus. Ann Phytopathol.

[ppat-0030008-b048] Moriones E, Díaz I, Rodríguez-Cerezo E, Fraile A, García-Arenal F (1992). Differential interactions among strains of tomato aspermy virus and satellite RNAs of *Cucumber mosaic virus*. Virology.

[ppat-0030008-b049] Escriu F, Perry KL, García-Arenal F (2000). Transmissibility of *Cucumber mosaic virus* by Aphis gossypii correlates with viral accumulation and is affected by the presence of its satellite RNA. Phytopathol.

[ppat-0030008-b050] Aranda MA, Fraile A, García-Arenal F, Malpica JM (1995). Experimental evaluation of the ribonuclease protection assay method for the assessement of genetic heterogeneity in populations of RNA viruses. Arch Virol.

[ppat-0030008-b051] Sambrook J, Russell DW (2001). Molecular cloning: A laboratory manual. 3rd edition.

[ppat-0030008-b052] Sokal RR, Rohlf FJ (1995). Biometry.

[ppat-0030008-b053] Devlin B, Risch N (1995). A comparison of linkage disequilibrium measures for fine-scale mapping. Genomics.

