# Supplementary material for: Constraints to Genetic Exchange Support Gene Coadaptation in a Tripartite RNA Virus
Source: PLoS Pathog. 2007 Jan 26;3(1):e8. doi: 10.1371/journal.ppat.0030008 (PMC1781478; doi:10.1371/journal.ppat.0030008)
Supplement: Table S1 — Data are number of descendants and frequencies referred to the total of the progeny (between brackets). Genotype distributions with the same letter did not differ at a 95% level of confidence. (72 KB DOC) [file ppat.0030008.st001.doc]

Table S1.- Frequency distribution^a^ of genetic types in progenies from double-inoculations of IA and IB CMV isolates on the local lesion host *Chenopodium quinoa* (LLH).

|  | Pair combinations of CMV isolates | | | | | | | |
| --- | --- | --- | --- | --- | --- | --- | --- | --- |
|  | I | | II | | III | | IV | |
| Genotypes^b^: | a | | b | | ab | | ab | |
| 1 AAA.A | 8 | (0.10) | 14 | (0.21) | 23 | (0.25) | 21 | (0.21) |
| 2 BAA.A | 29 | (0.37) | 24 | (0.36) | 22 | (0.25) | 24 | (0.25) |
| 3 ABA.A | 3 | (0.04) | 6 | (0.09) | 7 | (0.08) | 11 | (0.11) |
| 4 AAB.B | 0 |  | 6 | (0.09) | 2 | (0.02) | 6 | (0.06) |
| 5 BBA.A | 27 | (0.34) | 9 | (0.14) | 15 | (0.17) | 17 | (0.17) |
| 6 BAB.B | 4 | (0.05) | 1 | (0.02) | 5 | (0.06) | 9 | (0.09) |
| 7 ABB.B | 2 | (0.02) | 3 | (0.04) | 5 | (0.06) | 4 | (0.04) |
| 8 BBB.B | 6 | (0.08) | 3 | (0.04) | 10 | (0.11) | 7 | (0.07) |
| Recombinants in RNA3 | 0 |  | 0 |  | 0 |  | 0 |  |
| Allelic values: |  |  |  |  |  |  |  |  |
| *i* = A | 13 | (0.16) | 29 | (0.44) | 37 | (0.42) | 42 | (0.42) |
| *i* = B | 66 | (0.84) | 37 | (0.56) | 52 | (0.58) | 57 | (0.58) |
| *j* = A | 41 | (0.52) | 45 | (0.68) | 52 | (0.58) | 60 | (0.61) |
| *j* = B | 38 | (0.48) | 21 | (0.32) | 37 | (0.42) | 39 | (0.39) |
| *k*_1_.*k*_2_ = A | 67 | (0.85) | 53 | (0.80) | 67 | (0.75) | 73 | (0.74) |
| *k*_1_.*k*_2_ = B | 12 | (0.15) | 13 | (0.20) | 22 | (0.25) | 26 | (0.26) |
| Total (*N*) | 79 |  | 66 |  | 89 |  | 99 |  |
| Fitted model^c^: |  |  |  |  |  |  |  |  |
| α_A_ | -1.11135 ^**^ | | -0.12921 | | -0.18457 | | -0.16430 | |
| α_B_ | 0.51335 | | 0.11441 | | 0.15575 | | 0.14108 | |
| β_A_ | 0.03727 | | 0.31015 ^**^ | | 0.15575 | | 0.19237 ^*^ | |
| β_B_ | -0.03871 | | -0.45199 | | -0.18457 | | -0.23841 | |
| γ_A_ | 0.52839 ^**^ | | 0.47378 ^**^ | | 0.40920 ^**^ | | 0.38849 ^**^ | |
| γ_B_ | -1.19139 | | -0.93156 | | -0.70445 | | -0.64388 | |
| βγ_AA_ |  | |  | | 0.13936 ^**^ | |  | |
| βγ_AB_ |  | |  | | -0.60774 | |  | |
| βγ_BA_ |  | |  | | -0.23593 | |  | |
| βγ_BB_ |  | |  | | 0.49473 | |  | |

(^a^): Data are number of descendents and frequencies referred to the total of the progeny (between brackets). Genotype distributions with the same letter did not differ at a 95% level of confidence.

(^b^): Genotypes (*ijk*_1_.*k*_2_) are defined by the allelic value A (genetic type IA) or B (genetic type IB) at loci *i* (ORF 1a), *j* (ORF 2a) and *k*_1_.*k*_2_ (ORFs 3a and CP). The two later are presented together as no recombination was detected.

(^c^): Parameters in the log-linear model: Ln[*F*(*ijk*_1_.*k*_2_)] = Ln[*F_h_*(*ijk*_1_.*k*_2_)] + ϕ*_h_*, where ϕ*_h_* = α*_i_* + β*_j_* + γ_k_ + + αβ*_ij_* + αγ*_ik_* +βγ*_jk_* + αβγ*_ijk_* (see Materials and Methods) have been calculated from the random expectation hypothesis *F_h_*(ijk) = 0.125 · *N*. Significance of parameters at 95% or 99% levels of confidence is indicated by (^*^) or (^**^), respectively.
