# Supplementary material for: Constraints to Genetic Exchange Support Gene Coadaptation in a Tripartite RNA Virus
Source: PLoS Pathog. 2007 Jan 26;3(1):e8. doi: 10.1371/journal.ppat.0030008 (PMC1781478; doi:10.1371/journal.ppat.0030008)
Supplement: Table S2 — Progenies recovered from total RNA extracts of TIL. Data are number of descendants and frequencies referred to the total of the progeny (between brackets). Genotype distribution with the same letter did not differ at a 95% level of confidence. (65 KB DOC) [file ppat.0030008.st002.doc]

Table S2.- Frequency distribution^a^ of genetic types in progenies from double- inoculations of IA and IB CMV isolates on the systemic host *Nicotiana tabacum* cv. Xanthi-nc. Progenies recovered from total RNA extracts of inoculated leaves (TIL).

|  | Pair combinations of CMV isolates | | | | | | | |
| --- | --- | --- | --- | --- | --- | --- | --- | --- |
|  | I | | II | | III | | IV | |
| Genotype^b^: | a | | a | | b | | c | |
| 1 AAA.A | 44 | (0.75) | 74 | (0.55) | 11 | (0.12) | 14 | (0.18) |
| 2 BAA.A | 8 | (0.13) | 27 | (0.20) | 27 | (0.29) | 62 | (0.78) |
| 3 ABA.A | 4 | (0.07) | 16 | (0.12) | 12 | (0.13) | 0 |  |
| 4 AAB.B | 0 |  | 1 | (0.01) | 1 | (0.01) | 1 | (0.01) |
| 5 BBA.A | 3 | (0.05) | 12 | (0.09) | 39 | (0.42) | 0 |  |
| 6 BAB.B | 0 |  | 1 | (0.01) | 0 |  | 2 | (0.03) |
| 7 ABB.B | 0 |  | 1 | (0.01) | 0 |  | 0 |  |
| 8 BBB.B | 0 |  | 1 | (0.01) | 3 | (0.03) | 0 |  |
| Recombinants in RNA3 | 0 |  | 0 |  | 0 |  | 0 |  |
| Allelic value: |  |  |  |  |  |  |  |  |
| *i* = A | 48 | (0.81) | 92 | (0.69) | 24 | (0.26) | 15 | (0.19) |
| *i* = B | 11 | (0.19) | 41 | (0.31) | 69 | (0.74) | 64 | (0.81) |
| *j* = A | 52 | (0.88) | 103 | (0.77) | 39 | (0.42) | 79 | (1.00) |
| *j* = B | 7 | (0.12) | 30 | (0.23) | 54 | (0.58) | 0 |  |
| *k*_1_.*k*_2_ = A | 59 | (1.00) | 129 | (0.97) | 89 | (0.96) | 76 | (0.96) |
| *k*_1_.*k*_2_ = B | 0 |  | 4 | (0.03) | 4 | (0.04) | 3 | (0.04) |
| Total (*N*) | 59 |  | 133 |  | 93 |  | 79 |  |
| Fitted model^c^ |  |  |  |  |  |  |  |  |
| α_A_ | 0.48681 ^**^ | | 0.32459 ^**^ | | -0.66140 ^**^ | | -0.96825 ^**^ | |
| α_B_ | -0.98649 | | -0.48363 | | 0.39465 | | 0.48258 | |
| β_A_ | 0.56685 ^**^ | | 0.43753 ^**^ | | -0.17589 | | 0.69315 ^**^ | |
| β_B_ | -1.43848 | | -0.79600 | | 0.14953 | | -E | |
| γ_A_ | 0.69315 ^**^ | | 0.66261 ^**^ | | 0.64912 ^**^ | | 0.65443 ^**^ | |
| γ_B_ | -E | | -2.81091 | | -2.45316 | | -2.57769 | |

(^a^): Data are number of descendents and frequencies referred to the total of the progeny (between brackets). Genotype distributions with the same letter did not differ at a 95% level of confidence.

(^b^): Genotypes (*ijk*_1_.*k*_2_) are defined by the allelic value A (genetic type IA) or B (genetic type IB) at loci *i* (ORF 1a), *j* (ORF 2a) and *k*_1_.*k*_2_ (ORFs 3a and CP). The two later are presented together as no recombination was detected.

(^c^): Parameters in the log-linear model: Ln[*F*(*ijk*_1_.*k*_2_)] = Ln[*F_h_*(*ijk*_1_.*k*_2_)] + ϕ*_h_*, where ϕ*_h_* = α*_i_* + β*_j_* + γ_k_ + + αβ*_ij_* + αγ*_ik_* +βγ*_jk_* + αβγ*_ijk_* (see Materials and Methods) have been calculated from the random expectation hypothesis *F_h_*(ijk) = 0.125 · *N*. Letter E represents a trend to infinity. Significance of parameters at 95% or 99% levels of confidence is indicated by (^*^) or (^**^), respectively.
