# Supplementary material for: Constraints to Genetic Exchange Support Gene Coadaptation in a Tripartite RNA Virus
Source: PLoS Pathog. 2007 Jan 26;3(1):e8. doi: 10.1371/journal.ppat.0030008 (PMC1781478; doi:10.1371/journal.ppat.0030008)
Supplement: Table S3 — Progenies recovered from virion-encapsidated RNA from VIL. Data are number of descendants and frequencies referred to the total of the progeny (between brackets). Genotype distribution with the same letter did not differ at a 95% level of confidence. (71 KB DOC) [file ppat.0030008.st003.doc]

Table S3.- Frequency distribution^a^ of genetic types in progenies from double-inoculations of IA and IB CMV isolates on the systemic host *Nicotiana tabacum* cv. Xanthi-nc. Progenies recovered from virion-encapsidated RNA from inoculated leaves (VIL).

|  | Pair combinations of CMV isolates | | | | | | | |
| --- | --- | --- | --- | --- | --- | --- | --- | --- |
|  | I | | II | | III | | IV | |
| Genotype^b^: | a | | b | | c | | b | |
| 1 AAA.A | 14 | (0.29) | 47 | (0.55) | 37 | (0.38) | 43 | (0.56) |
| 2 BAA.A | 25 | (0.51) | 33 | (0.38) | 25 | (0.25) | 32 | (0.42) |
| 3 ABA.A | 0 |  | 3 | (0.03) | 8 | (0.08) | 0 |  |
| 4 AAB.B | 1 | (0.02) | 1 | (0.01) | 0 |  | 1 | (0.01) |
| 5 BBA.A | 9 | (0.18) | 3 | (0.03) | 27 | (0.28) | 0 |  |
| 6 BAB.B | 0 |  | 0 |  | 0 |  | 1 | (0.01) |
| 7 ABB.B | 0 |  | 0 |  | 0 |  | 0 |  |
| 8 BBB.B | 0 |  | 0 |  | 1 | (0.01) | 0 |  |
| Recombinants in RNA3 | 0 |  | 0 |  | 0 |  | 0 |  |
| Allelic value: |  |  |  |  |  |  |  |  |
| *i* = A | 15 | (0.31) | 51 | (0.59) | 45 | (0.46) | 44 | (0.57) |
| *i* = B | 34 | (0.69) | 36 | (0.41) | 53 | (0.54) | 33 | (0.43) |
| *j* = A | 40 | (0.82) | 81 | (0.93) | 62 | (0.63) | 77 | (1.00) |
| *j* = B | 9 | (0.18) | 6 | (0.07) | 36 | (0.37) | 0 |  |
| *k*_1_.*k*_2_ = A | 48 | (0.98) | 86 | (0.99) | 97 | (0.99) | 75 | (0.97) |
| *k*_1_.*k*_2_ = B | 1 | (0.02) | 1 | (0.01) | 1 | (0.01) | 2 | (0.03) |
| Total (*N*) | 49 |  | 87 |  | 98 |  | 77 |  |
| Fitted model^c^ |  |  |  |  |  |  |  |  |
| α_A_ | -0.49062 ^**^ | | 0.15906 | | -0.08516 | | 0.13353 | |
| α_B_ | 0.32769 | | -0.18924 | | 0.07847 | | -0.15415 | |
| β_A_ | 0.49021 ^**^ | | 0.62169 ^**^ | | 0.23531 ^*^ | | 0.69315 ^**^ | |
| β_B_ | -1.00145 | | -1.98100 | | -0.30830 | | -E | |
| γ_A_ | 0.67253 ^**^ | | 0.68159 ^**^ | | 0.68289 ^**^ | | 0.66683 ^**^ | |
| γ_B_ | -3.19867 | | -3.77276 | | -3.89182 | | -2.95751 | |
| αβ_AA_ | 0.20294 ^*^ | |  | | 0.26209 ^**^ | |  | |
| αβ_AB_ | -E | |  | | -0.72577 | |  | |
| αβ_BA_ | -0.10454 | |  | | -0.29358 | |  | |
| αβ_BB_ | 0.36546 | |  | | 0.36336 | |  | |

(^a^): Data are number of descendents and frequencies referred to the total of the progeny (between brackets). Genotype distributions with the same letter did not differ at a 95% level of confidence.

(^b^): Genotypes (*ijk*_1_.*k*_2_) are defined by the allelic value A (genetic type IA) or B (genetic type IB) at loci *i* (ORF 1a), *j* (ORF 2a) and *k*_1_.*k*_2_ (ORFs 3a and CP). The two later are presented together as no recombination was detected.

(^c^): Parameters in the log-linear model: Ln[*F*(*ijk*_1_.*k*_2_)] = Ln[*F_h_*(*ijk*_1_.*k*_2_)] + ϕ*_h_*, where ϕ*_h_* = α*_i_* + β*_j_* + γ_k_ + + αβ*_ij_* + αγ*_ik_* +βγ*_jk_* + αβγ*_ijk_* (see Materials and Methods) have been calculated from the random expectation hypothesis *F_h_*(ijk) = 0.125 · *N*. Letter E represents a trend to infinity. Significance of parameters at 95% or 99% levels of confidence is indicated by (^*^) or (^**^), respectively.
